# A bi-directional dialog between vascular cells and monocytes/macrophages regulates tumor progression

**DOI:** 10.1007/s10555-021-09958-2

**Published:** 2021-03-30

**Authors:** Victor Delprat, Carine Michiels

**Affiliations:** Biochemistry and Cellular Biology Research Unit (URBC), Namur Research Institute for Life Sciences (NARILIS), University of Namur (UNamur), 61 Rue de Bruxelles, B-5000 Namur, Belgium

**Keywords:** Cancer, Endothelial cell, Pericyte, Monocyte/macrophage, Angiogenesis, Metastasis

## Abstract

Cancer progression largely depends on tumor blood vessels as well on immune cell infiltration. In various tumors, vascular cells, namely endothelial cells (ECs) and pericytes, strongly regulate leukocyte infiltration into tumors and immune cell activation, hence the immune response to cancers. Recently, a lot of compelling studies unraveled the molecular mechanisms by which tumor vascular cells regulate monocyte and tumor-associated macrophage (TAM) recruitment and phenotype, and consequently tumor progression. Reciprocally, TAMs and monocytes strongly modulate tumor blood vessel and tumor lymphatic vessel formation by exerting pro-angiogenic and lymphangiogenic effects, respectively. Finally, the interaction between monocytes/TAMs and vascular cells is also impacting several steps of the spread of cancer cells throughout the body, a process called metastasis. In this review, the impact of the bi-directional dialog between blood vascular cells and monocytes/TAMs in the regulation of tumor progression is discussed. All together, these data led to the design of combinations of anti-angiogenic and immunotherapy targeting TAMs/monocyte whose effects are briefly discussed in the last part of this review.

## Introduction

### Tumor-associated macrophages

#### TAMs in the tumor microenvironment

Tumor-associated macrophages (TAMs) are major tumor microenvironment (TME) cells and represent an important part of the cancer immune infiltrate. TAM infiltration and TAM numbers are correlated with poor prognosis in a majority of cancer types [1]. TAMs play an important role in cancer development notably via the promotion of tumor growth, tumor inflammation, angiogenesis, lymphangiogenesis, metastasis, immunosuppression, and chemotherapeutic resistance [2–5]. Macrophages are classified as pro-inflammatory M1 macrophages and anti-inflammatory M2 macrophages. This classification is oversimplified since TAMs can express both M1 and M2 markers, and hence, TAMs are classified onto a M1 and M2 polarization axis in which M1 and M2 macrophages are the two extremes. Basically, and based on *in vitro* experiments, M1 macrophages are polarized with pro-inflammatory cytokines and bacterial molecules such as interferon γ and lipopolysaccharides. M1 macrophages express high levels of pro-inflammatory cytokines (e.g*.*, IL-12, tumor necrosis factor α (TNFα), and IL-6) and intracellular host response genes (e.g., CD80 and IFIT1) [6, 7]. M2 macrophages are divided into at least three subsets called M2a, M2b, and M2c. This M2 classification in three subsets was firstly proposed in [6]. M2a are activated by IL-4 and/or IL-13 and express high levels of CD206, CD163, and fibronectin [6, 7]. M2b are induced by Toll-like receptors ligands and immune complex activation, whereas M2c are activated by IL-10. Interestingly, macrophage M2 polarization is also induced by the TME [8–14]. Nonetheless, the three classes of M2 macrophages share common features such as IL-12^low^ and IL-10^high^ and arginase-1 (Arg-1)^high^, whereas M1 macrophages are IL-12^high^, IL-23^high^, and IL-10^low^. In the TME, CD163 and CD206 are commonly used to identify macrophages from the M2 population, whereas CD86 is a common M1 marker.

#### Origins of TAMs

There exist at least two origins of TAMs. TAMs can originate either from tissue-resident macrophages (TRMs) or from blood vessel inflammatory monocytes (IMs) CCR2^+^, which are recruited via CCL2 chemotaxis [15–17]. TRMs are present in healthy tissues, hence before cancer initiation [15]. TRMs arise from embryonic progenitor–derived macrophages (e.g., brain macrophages also called microglia) or from blood monocytes (e.g., intestine or dermis). Furthermore, TRMs are able to self-maintain without adult blood monocyte contribution. Although TRMs are known for a while, the implication of TRMs in cancers has only recently been investigated, mostly in murine tumor models. For example, TRMs promote pancreatic ductal adenocarcinoma (PDAC) progression [18]. Indeed, colony-stimulating factor 1 (CSF1) antibodies combined with clodronate liposome followed by 10 days of blood monocyte recovery induce an almost complete TRM depletion without affecting circulating monocyte. In these conditions, tumor burden and high-grade carcinoma development are drastically reduced [18]. Nonetheless, monocyte-derived macrophages represent the major macrophage population in a majority of murine cancer types, such as breast, lung, brain, and hepatocellular carcinoma [15]. Monocytes are classified into 3 subsets in humans and in mice, according to marker expression [19, 20]. There are IMs (CD16^−^/CD14^+^/CX_3_CR1^lo^ in human, Ly6C^high^/CD43^lo^/CX3CR1^lo^ in mouse), non-classical monocytes (or patrolling, CX3CR1^high^, CD14^lo^, CD16^+^ in human and Ly6C^lo^/CD43^high^/CX3CR1^high^ in mouse), and intermediate monocytes (CX3CR1^high^, CD14^+^, CD16^+^ in human, Ly6C^int^CD43^hi^CX3CR1^hi^ in mouse). Numerous murine studies showed that IMs are the major source of TAMs in tumors, such as mammary tumors and their associated lung metastases, hepatocellular carcinoma, orthotopic Lewis lung carcinoma (LLc), and PDAC [19]. Furthermore, IMs display pro-tumoral functions such as angiogenesis and metastasis promotion [19]. Non-classical and intermediate monocytes have pro and anti-tumoral functions. Indeed, human intermediate and non-classical CD16^+^ monocytes promote angiogenesis *in vitro* [21] and murine Ly6G^lo^ patrolling monocytes are immunosuppressive in vivo [22, 23], whereas murine patrolling monocytes prevent breast to lung metastasis in murine PyMT breast cancer model [24]. Another type of monocyte classification exists, based on the receptor tyrosine kinase Tie2 expression. Indeed, recently, Tie2-expressing monocytes (TEMs) have been discovered by De Palma and colleagues [25, 26]. Before these studies, only ECs were thought to express the angiopoietin (1–4) receptor Tie2 [25]. Nowadays, some cell types have been discovered to express Tie2: endothelial cells (ECs), TEMs, a subset of TAMs, pericyte precursors of mesenchymal origin, a subset of hematopoietic stem cells, and some cancer cell lines [26–28]. Two studies showed that Tie2 is expressed mainly by intermediate monocyte (CD14^+^ CD16^+^), whereas one study shows that Tie2 is also expressed in non-classical monocyte (CD14^dim^ CD16^+^). Hence, Tie2 is expressed mostly but not exclusively in CD16^+^ monocytes and to a lesser extent in CD16^−^ monocytes [29].

### Tumor blood vessels

#### The onset of angiogenesis or the “angiogenic switch”

During cancer development, the transition from an avascular tumor to a vascularized tumor, called the “angiogenic switch” is a critical step [30, 31]. This switch occurs when the balance between pro-angiogenic factors (e.g., vascular endothelial growth factor (VEGF)-A) and anti-angiogenic factors (e.g., statins) shifts towards angiogenesis [30]. This switch appears during the progression from hyperplasia to neoplasia and coincides with malignant transition in PyMT and RIP1-Tag2 mice models. It is needed for malignant tumor progression [32, 33]. Immune cells such as TAMs and neutrophils are involved in this process. For example, in the PyMT murine breast cancer model, high TAM infiltration precedes the onset of angiogenesis. Furthermore, vasculature development is observed earlier in this model when macrophage infiltration is induced with CSF1 transgenic overexpression specifically in mammary tissues [32]. In the Rip1-Tag2 mouse pancreatic tumor model, neutrophil ablation with anti-Gr1 antibody strongly diminishes tumor vessel development [34].

#### Lymphatic vasculature and lymphangiogenesis

Lymphatic vasculature is critically involved in fluid homeostasis regulation, immune cell dissemination/surveillance, and lipid reabsorption. Absence or non-functional lymphatic system causes lymphedema, a disease characterized by huge swelling and repeated skin infections.

Lymphangiogenesis is defined as the formation of new lymphatic vessels from existing ones. It occurs during embryonic development and during tumor growth. It is correlated with a bad prognosis in cancer [35]. Lymphatic vessel hyaluronic receptor 1 (LYVE 1), podoplanin, and prospero homeobox 1 (prox1) are lymphatic EC (LEC) markers. Mechanistically, VEGF-C and VEGF-D are the two main lymphangiogenic factors which promote lymphangiogenesis by activating LECs VEGF receptor 3 (VEGFR-3) [5]. Lymphatic vasculature is critically involved in the metastatic spread of cancer cells into lymph nodes and finally to distant organs [36–39]. Lymphatic vessel density and lymph node status (i.e., the presence or the absence of cancer cells) is associated with poor prognosis and metastasis in several cancers [35]. The link between VEGF-C, VEGF-D, lymphatic vessel density, lymph node metastasis, and prognosis is extensively reviewed in [40].

#### Tumor blood vessels and immune system: endothelial anergy

During cancer progression, the immune system is progressively modified by the TME in a process called immunoediting. This process is composed of three phases, namely elimination, equilibrium, and escape. In the two first phases, the immune system is able to kill cancer cells notably via CD8^+^ T cells and natural killer (NK) cells. During these stages, TAMs belong mostly to M1 phenotype and are able to kill cancer cells and to activate the immune system. For example, in early-stage human lung tumors, TAMs mostly share both M1 and M2 markers and are able to activate T cell function, and hence are anti-tumoral [41]. In pancreatic pre-cancerous lesions, in gastrointestinal stromal tumors, in ovarian cancer, and in bladder cancer, TAMs mostly belong to the M1 phenotype and are progressively skewed toward the M2 phenotype during disease progression [42–44]. In later stages, TAMs display mostly M2 phenotype and are pro-tumoral and immunosuppressive.

Tumor blood vessels constitute a barrier regulating immune cell recruitment from blood into tumor via extravasation. The regulation of immune cell extravasation into tumor through blood vessels is then crucial in the regulation of tumor progression. This process is highly regulated and is composed of several steps. First, there is leukocyte rolling followed by the arrest and firm adhesion to ECs. Then, leukocytes transmigrate through ECs to extravasate and infiltrate the tissue. This process requires adhesion molecules expressed by ECs such as E-selectin (rolling), ICAM1 and VCAM1 (firm arrest), and VE-cadherin and CD31 (transendothelial migration) [45]. The expression of these proteins is tightly regulated and promoted by inflammatory stimuli such as TNFα. Tumor vessels are modified by TME to induce endothelial anergy, notably via VEGF [46]. In this state, tumor endothelial cells are unresponsive to pro-inflammatory stimuli such as TNFα and hence do not promote anymore leukocyte extravasation [47]. This anergy is crucial in tumor growth promotion, likely more importantly during the elimination and equilibrium phases, because the immune system is anti-tumoral. For example, the overexpression of EGF-like domain–containing protein 7 (Egfl7) in cancer cells, an endothelial activation repressor [48], promotes tumor growth and development by preventing leukocyte infiltration via endothelial E-selectin and ICAM1 and VCAM1 adhesion molecule repression [49].

Tumor blood and lymphatic vessels also modulate the immune system (this is well reviewed in [46]). Indeed, lymphatic ECs (LECs) and ECs both express program death-ligand 1 (PD-L1), which inhibits T cell function [50, 51]. Furthermore, ECs can induce T cell apoptosis by Fas ligand expression [52]. Tumor ECs are modified by the TME. Indeed, IL-6 and IL-10 secretion from lung tumor ECs is strongly increased. Normal lung ECs induce strong NK cell activation, whereas this ability is strongly reduced in ECs from lung tumors [53]. Furthermore, IL-6 and IL-10 cytokines are involved in macrophage polarization towards M2 phenotype and hence promote tumor growth [54, 55].

To recapitulate, tumor blood vessels regulate immune cell infiltration as well as their activation in tumors. In this review, the impact of vascular cells (ECs and pericytes) on monocyte and TAM recruitment into tumors will be discussed. Furthermore, the impact of vascular cells on monocyte and TAM angiogenic phenotype and polarization will also be described. Reciprocally, the impact of TAMs, TEMs, and classical and non-classical monocytes on blood vessels will be emphasized. Their impact on angiogenesis, lymphangiogenesis, and metastasis will be detailed.

## Effects of vascular and perivascular cells on macrophages (related to Fig. 1)

### Monocyte and macrophage recruitment by ECs and pericytes (related to Fig. 1a)

TAM recruitment in cancer is involved in the angiogenic switch induction; promotes tumor growth, metastasis, vessel “abnormalization”; and is associated with a bad prognosis in most cancer types. Indeed, macrophage depletion by different ways has a negative impact on these features. TAMs are recruited by different chemokines and cytokines such as chemokine (C-C motif) ligand 2 (CCL2), CCL5, CCL7, angiopoietin-2 (Ang-2), CSF1, VEGF, IL-33, semaphorin 3D, endothelial monocyte–activating polypeptide-II (EMAP-II), endothelin (ET)-1 and 2, stromal cell–derived factor 1α (SDF1α/CXCL12), eotaxin, and oncostatin which are secreted by cancer cells, stromal cells, and perivascular and vascular cells. This is extensively reviewed in [69, 70]. TAMs are classified not only according to their marker expression into M1 or M2 phenotype, but also according to their tumor localization into migratory TAMs or perivascular TAMs [4, 17]. Here, we will focus on the effects of ECs and perivascular cells on TAM and monocyte recruitment as well as on their localization within the tumor.

**Fig. 1:**
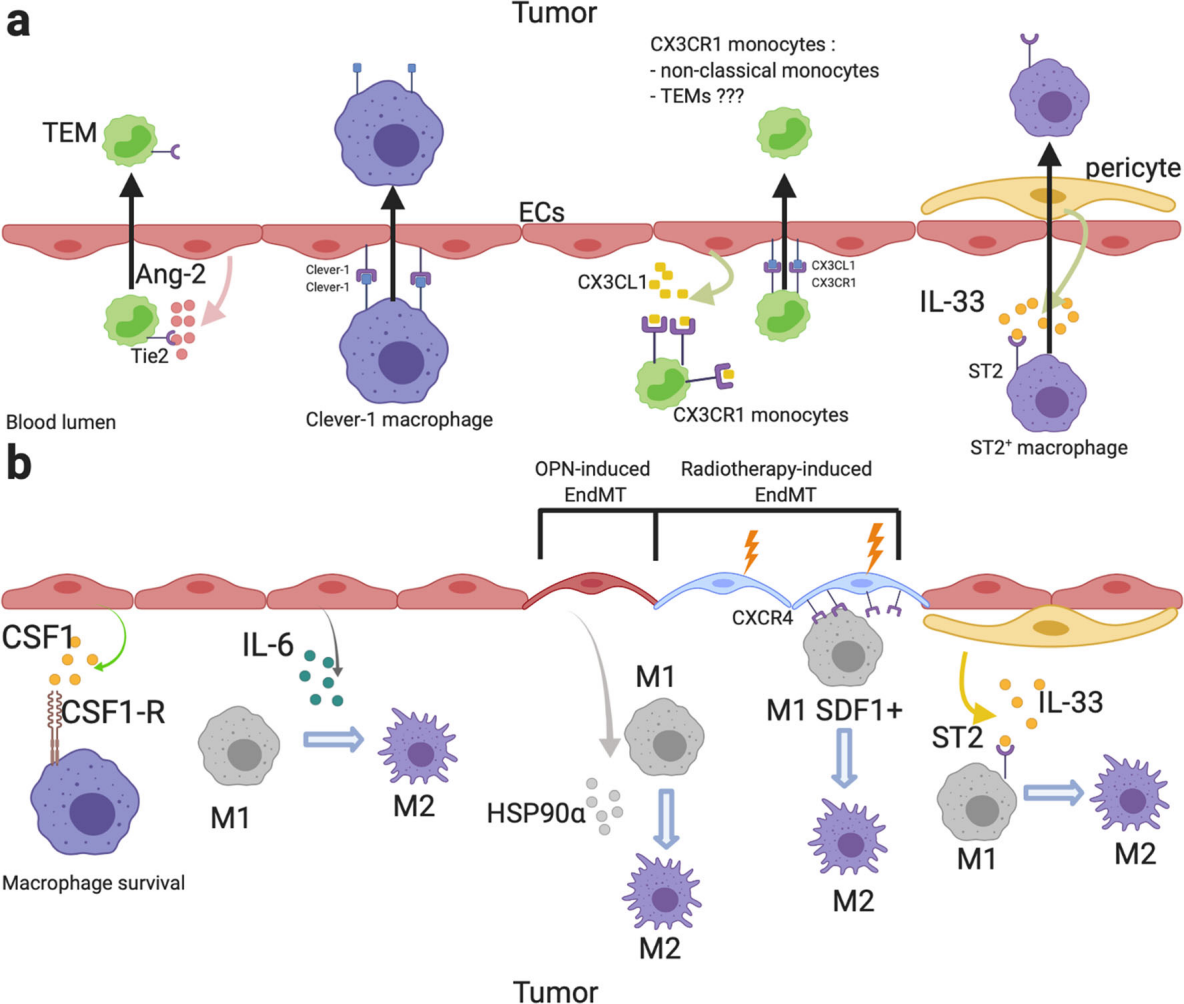
Effects of blood vessels on monocyte/macrophage recruitment and polarization. **a** Effects of ECs and pericytes on monocyte/macrophage recruitment. ECs secrete high dose of Ang-2 which induces TEM recruitment in tumor [56–59]. Furthermore, Ang-2 promotes angiogenic phenotype in TEMs and in Tie2-expressing macrophages [58]. Homophilic interaction between Clever-1 in ECs and TAMs induces TAM infiltration [60]. ECs secrete CX3CL1 which induces CX3CR1-expressing monocyte (non-classical monocyte) chemotaxis toward ECs [21, 61]. CX3CL1/CX3CR1 interaction induces non-classical monocyte recruitment via VEGF-A-dependent CX3CL1 shedding [21, 62, 63]. IL-33 secreted by pericytes promotes TAM recruitment via the IL-33 receptor ST2 activation [64]. **b** Effects of ECs and pericytes on TAM survival and polarization. CSF1 promotes TAM survival in the TME via CSF1R activation [65]. ECs are high IL-6 producer. EC-derived IL-6 induces TAM M2 polarization [12]. Osteopontin-induced EndMT promotes M2 TAMs polarization via HSP90⍺ secretion [66]. Radiotherapy-induced EndMT induces CXCR4 expression in ECs, which promotes SDF1α-expressing TAM M2 polarization [67]. IL-33 secreted by pericytes promotes M2 polarization in a ST2-dependent manner [68]. This figure was created with BioRender.com

#### EC-derived angiopoietin-2 (Ang-2)

Ang-2 is mainly released by ECs in tumors, but in some cases, Ang-2 is also expressed by cancer cells [71]. Ang-2 is stored in Weibel-Palade bodies in ECs [72, 73], and its expression and release from EC are regulated by CTHRC1/ERK/AP-1 signaling and by neuroligin 2 [74, 75]. *In vitro*, EC-derived Ang-2 induces chemotaxis of Tie2^+^ macrophages and monocytes. THP-1 Tie2^+^ monocytes but not Tie2^−^ migrate towards Ang-2 in the Boyden chamber model [76]. U937 monocytes exposed to Kaposi’s sarcoma EC conditioned media migrate towards the conditioned medium compartment. This migration is abolished with anti-Ang-2 antibody or with Ang-2 shRNA in ECs [56]. Hence, Ang-2 expression and release by EC are tightly regulated and promote TEM migration *in vitro.*

*In vivo*, Ang-2 induces Tie2^+^ TAM and TEM infiltration by stimulating the expression of their Ang-2 receptor Tie2. Indeed, Ang-2 induces macrophage and TEM infiltration that is correlated with metastasis in murine MDA-MB-231-induced breast cancer, in pancreatic cancers, in lung cancer, in Kaposi’s sarcoma, in glioblastomas (GBMs), and in gliomas [56–58, 74, 77]. Indeed, specific EC Ang-2 overexpression increases macrophage and TEM infiltration in murine GBM and LLc lung tumor models [57, 58]. Ang-2 inhibition diminishes TAM and/or TEM infiltration in Kaposi’s sarcoma and breast cancer murine models [56, 59]. Nonetheless, in MMTV-PyMT breast cancer and Rip1-Tag2 pancreatic cancer, Ang-2 inhibition does not modify macrophage or TEM infiltration but rather inhibits their perivascular localization [78]. Ang-2 blockade induces SDF1α overexpression in the MMTV-PyMT model, which can counterbalance the effects of Ang-2 blockade on TAM and TEM infiltration. Ang-2 induces EC ICAM1 and VCAM1 expression that hence increases monocyte and TAM adhesion on EC [77]. Moreover, Ang-2 increases vessel permeability, angiogenesis, and CCL2 expression in ECs that also leads to C-C chemokine receptor type 2 (CCR2)^+^ monocyte and TAM infiltration [77, 79]. Hence, in tumors, Ang-2 is an important EC-secreted protein that is involved in macrophage and monocyte Tie2^+^ infiltration and perivascular localization. Moreover, Ang-2-induced EC CCL2 overexpression induces CCR2^+^ IM recruitment.

In line with the fact that Ang-2 induces TAM and TEM infiltration, Ang-2 expression is correlated with microvascular density and associated with poor prognosis in several cancers [71]. Furthermore, Ang-2 is overexpressed in tumor tissues compared to normal tissues [71]. Ang-2 expression is increased by anti-VEGF therapies in tumor but not in normal tissues [76, 80–82]. This Ang-2 overexpression leads to therapy failure by increasing TEM and TAM infiltration. This TAM recruitment induced by anti-VEGF therapy is blocked by the addition of Ang-2 antibody or soluble Tie2 [76]. This bitherapy has been tested in phase I in human cancer patients and showed acceptable safety and encouraging antitumor activity [83]. To summarize, Ang-2 is involved in tumor resistance against VEGF therapy and anti-Ang-2/VEGF combination shows encouraging results in pre-clinical and clinical studies.

#### Pericytes and perivascular cancer-associated fibroblasts in TAM recruitment

Pericytes and perivascular cancer-associated fibroblasts (CAFs) are involved in TAM recruitment and their perivascular localization. Platelet-derived growth factor (PDGF)-BB secretion by cancer cells induces IL-33 expression and secretion by pericytes and CAFs via a PDGF receptor β (PDGFRβ)–dependent mechanism. IL-33 further stimulates macrophage migration *in vitro* and TAM infiltration *in vivo* via the IL-33 receptor ST2–dependent mechanism [64]. Indeed, pericytes exposed to PDGF-BB *in vitro* or *in vivo* in lung tumor model with LLc overexpressing PDGF-BB overexpress IL-33 and this overexpression is abolished by anti-PDGFRβ antibodies. IL33-induced RAW cell migration is abolished by ST2 RAW siRNA. *In vivo*, TAM infiltration is increased in tumors overexpressing PDGF-BB. This increase is abolished in mice IL-33^−/−^, ST2^−/−^ or with ST2 soluble factors. These IL-33 recruited TAMs are also involved in tumor growth and in cancer cell stemness via prostaglandin 2 secretion [84]. Milk fat globule-epidermal growth factor 8 (MFG-E8), expressed mostly by pericytes in melanoma tumors, is also involved in TAM infiltration by an unknown mechanism which would be interesting to clarify [85]. Consistently, high MFG-E8 expression is associated with high TAM infiltration in bladder cancer [86]. As said above, TAMs are also classified according to their tumor localization in which there are migratory TAMs and perivascular TAMs [4, 17]. In fact, there is a unidirectional mechanism by which a newly recruited monocyte will differentiate in migratory TAMs which then will be recruited to blood vessels and hence become perivascular [17]. Indeed, in the mammary PyMT model, newly tumor-infiltrated blood CCR2+ monocytes are recruited by cancer cell– and stromal cell–derived CCL2. Then, monocytes differentiate into migratory TAMs and C-X-C chemokine receptor type 4 (CXCR4) expression by TAMs is then promoted by tumor-derived transforming growth factor-β (TGF-β). These migratory TAMs are then recruited near to the blood vessel by SDF1α-derived perivascular CAFs [17]. In summary, perivascular cells are involved in TAM recruitment via IL-33 secretion and in TAM perivascular localization via SDF1α secretion.

#### Monocyte/TAM recruitment via direct interactions with ECs

Whereas most endothelial-leukocyte adhesion molecules are shared between all leukocyte types [46], monocytes and TAMs are also specifically recruited by tumor ECs [46]. Clever-1/stabilin-1^+^ is a scavenger receptor and an adhesion molecule regulating macrophage and T regulator lymphocyte transendothelial migration as well as tumor infiltration [60, 87]. Indeed, Clever-1 overexpressing ECs are involved in Clever-1^+^ monocyte/macrophage and Treg recruitment [60]. Indeed, Clever-1 deletion in mice or specifically in macrophages or in ECs leads to a diminished TAM recruitment, without affecting lymphocyte CD4^+^ or CD8^+^ recruitment [60]. Apoptosis signal-regulating kinase 1 (ASK1), a factor involved in the regulation of EC activation, is involved in TAM recruitment in tumor, without affecting lymphocyte CD3^+^ recruitment [88, 89]. In non-inflammatory conditions, ASK1 is consistently degraded via suppressor of cytokine signaling 1 (SOCS1) by the proteasome and pushes ECs in an inactivated state. In inflammatory condition, ASK1 is stabilized and stimulates EC activation via JNK/p38MAPK activation [89]. EC ASK1 expression induces macrophage infiltration into tumors without affecting lymphocyte recruitment [88]. TAM infiltration in tumors is decreased in ASK1 KO mice or with ASK1 inhibition specifically in EC (via SOCS1 overexpression specifically in EC) or with ASK1 inhibitor. This TAM infiltration prevention by ASK1 inhibition leads to a decrease in tumor growth and in metastasis and to an increased survival in mice [88]. Nonetheless, the mechanism by which ASK1 leads to specific TAM infiltration remains unclear and it would be interesting to be investigated. That could be either by chemotactic factor over-secretion specifically inducing TAM infiltration (e.g., CCL2, Ang-2) or via a direct contact between TAMs and ECs inducing TAM transmigration (e.g., via Clever-1 interaction). *In vitro*, TAM transmigration is impaired across EC ASK1–specific inhibition, but the lymphocyte transmigration has not been investigated [88]. All these data demonstrate that homophylic interaction between EC and macrophage Clever-1/stabilin-1 is involved in TAM recruitment into tumor without affecting CD4^+^ or CD8^+^ lymphocyte recruitment.

#### CX3CL1/CX3CR1 axis in non-classical monocyte recruitment

Chemokine (C-X3-C motif) ligand 1 (CX3CL1) expression in ECs is involved in the CX3CL1 receptor (CX3CR1)–dependent recruitment of immune cells, such as NK cells, CD8^+^ T cell, and CX3CR1 non-classical monocytes [90]. CX3CL1 expression in EC specifically regulates CX3CR1-expressing monocyte recruitment into tumors without affecting IM recruitment. This process may be also involved in TEM recruitment since around 50% of TEMs express CX3CR1 [29]. CX3CL1 exists as membrane bound and soluble forms. Soluble CX3CL1 is involved in CX3CR1 monocyte chemotaxis, whereas membrane bound is involved in their adhesion to ECs [61, 91]. Indeed, soluble CX3CL1 induces human peripheral blood mononuclear cell–derived monocyte migration, more effectively than CCL5 [91]. CX3CL1^+^ monocytes adhere to HEK293 overexpressing membrane bound CX3CR1 but not to WT HEK293. The membrane-bound CX3CL1 promotes human non-classical monocyte crawling and adhesion on endothelium via CX3CR1 activation on non-classical monocytes [21]. The subsequent monocyte transmigration is promoted by angiogenic factors such as VEGF-A. VEGF-A involvement in non-classical monocyte transmigration is due to VEGF-A-induced a disintegrin and metalloproteinase domain–containing protein 10 (ADAM10) and ADAM17 activity stimulation [62, 63], which subsequently promotes CX3CR1 monocyte transmigration via CX3CL1 shedding [92] and hence non-classical monocyte transmigration. Consistently with these results, *in vitro* transendothelial migration and *in vivo* infiltration of non-classical monocytes into tumors are critically lower in non-angiogenic tumors, whereas they are increased in angiogenic tumors [21]. Indeed, human non-classical monocytes are recruited mostly in DLD1 or HCT116 tumor expressing high level of VEGF-A, whereas they are less recruited in SKBR1 tumor expressing low level of VEGF-A. Furthermore, treatment of DLD1 tumors with anti-VEGF-A antibody bevacizumab reduces the human CD16^+^ monocyte recruitment. Nonetheless, the DC101 anti-VEGFR2 antibody increases Ly6C^lo^ monocyte infiltration into orthotopic murine colorectal tumors. Furthermore, non-classical monocytes require CX3CR1 to infiltrate tumors since Ly6C^lo^ monocytes infiltration in murine orthotopic colorectal tumors is abolished in CX3CR1 KO mice [22]. These data suggest that the interaction between CX3CL1 (EC) and CX3CR1 (non-classical monocyte) promotes non-classical monocyte recruitment into tumor. This recruitment is enhanced in angiogenic tumors and it would be interesting to investigate if this process is involved in TEM infiltration since 50% of TEMs express CX3CR1.

### Impact of blood vessel cells on macrophage polarization and angiogenic phenotype (related to Fig. 1b)

#### ECs promote M2 polarization and angiogenic phenotype

ECs are involved in macrophage survival, proliferation, M2-polarization, and angiogenic phenotype acquisition in malignant and non-malignant tissues [12, 54, 65]. The impact of ECs on macrophage survival has been demonstrated by co-culture experiments. The macrophage survival and expansion are mediated by direct contact between ECs and macrophages since macrophage colony formation is observed with direct co-cultures but not with transwell assays. CSF1-membrane bound (EC) and CSF1 receptor (CSF1R) (macrophage) juxtacrine interaction is involved in macrophage survival and expansion, since a CSF1 exclusive inhibitor inhibits macrophage survival and expansion [65]. This survival/proliferation induced by ECs in macrophages is likely due to mechanistic target of rapamycin (mTOR) activation in macrophage since mTOR inhibition with rapamycin inhibits CSF1+IL-6-induced macrophage proliferation [12]. Furthermore, ECs induce M2 polarization *in vitro* and *in vivo*, notably via IL-6 secretion [12, 54]. Indeed, the macrophage-EC co-cultures increase M2 marker expression such as Tie2 and decrease M1 marker expression such as major histocompatibility complex II (MHCII) [65]. Furthermore, EC conditioned media induce M2 polarization associated with the enhanced expression of CD206 or Arg-1, which is reduced by anti-IL-6 antibody [12]. This M2 polarization is enhanced in pre-incubated ECs with GBM cells which seems that this EC-induced M2 polarization is amplified by the TME. In human and murine GBM, alternatively activated TAMs are localized proximately to ECs, which are a major source of IL-6. Indeed, *in vivo*, specific inducible deletion of IL-6 in ECs reveals that ECs are the major source of IL-6 in murine GBM [12]. Furthermore, IL-6 expression is highly detected in ECs cytoplasm of newly formed vessels in human GBM [93]. Specific inducible deletion of IL-6 in ECs strongly decreases M2 macrophage population and slightly increases M1 population, decreases tumor growth, and enhances mice survival [12]. In summary, ECs are involved in macrophage survival and expansion via CSF1-CSF1R juxtacrine loop, and in macrophage M2 polarization, notably via IL-6 secretion *in vivo*, at least in murine GBM.

ECs induce angiogenic phenotype in macrophages associated with an increase in Tie2 or VEGF-A expression and macrophages co-cultivated with ECs increase murine prostate tumor growth and angiogenesis, when these macrophages are co-injected with cancer cells in mice [65]. EC-derived Ang-2 is not only a chemoattractant for TEMs. Indeed, Ang-2 also promotes M2 polarization and angiogenic profile in TEMs by increasing the expression of M2 markers (IL-10 and MRC1) and of angiogenesis-related gene (cathepsin B and thymidine phosphorylase) expression [58]. Furthermore, *in vivo*, Ang-2 and Ang-2 + VEGF inhibitions shift macrophage population from M2 towards M1. Anti-Ang-2 increases M1/M2 intermediate macrophage population in murine GBM. Anti-Ang-2 combined with an anti-VEGF increases M1 proportion among total leukocytes in the PyMT model and increases M1 population and decreases M2 population among total macrophages [81, 94]. Hence, Ang-2 is involved in macrophage and TEM M2 polarization and promotes their angiogenesis phenotype.

#### Endothelial-to-mesenchymal transition (EndMT) promotes macrophage M2 polarization

Endothelial-to-mesenchymal transition (EndMT) is defined as a phenotypic change in ECs characterized by a loss of endothelial features, markers (e.g., CD31), cellular tight junctions, apico-basal polarity, and the acquisition of mesenchymal features and markers such as fibroblast specific protein-1 and α-smooth muscle actin (α-SMA) [95]. EndMT is a source of up to 40% of CAFs; can be induced by radiotherapy, TGFβ-1, or osteopontin; and has an impact on tumorigenesis, metastatic extravasation, and therapy resistance [67, 95–98]. Radiotherapy-induced EndMT is mediated via p53 activation in ECs, whereas it is mediated via transcription factor 12 (TCF12) in osteopontin-induced EndMT since p53 siRNA and TCF12 shRNA inhibit radiotherapy-induced EndMT and osteopontin-induced EndMT, respectively. Furthermore, ECs undergoing EndMT with osteopontin or radiotherapy induce M2 polarization and inhibit M1 polarization [66, 67]. This is mediated via heat shock protein 90 α (HSP90α) secretion by osteopontin-induced EndMT, whereas it is mediated via CXCR4/SDF1α signaling in radiotherapy-induced EndMT [66, 67]. *In vitro*, osteopontin-induced EndMT conditioned media induce THP-1-derived macrophage M2 polarization which is blocked by anti-HSP90α antibody. On the other hand, bone marrow–derived macrophages (BMDMs) co-cultivated with irradiated tumor ECs display an increased CD206^+^ M2 macrophage proportion (in total F4/80^+^ macrophage population) compared with non-irradiated ECs. This effect is abolished in BMDMs co-cultivated with tumor ECs from EC-p53 KO mice. Furthermore, *in vivo*, subcutaneous co-injection of osteopontin-induced EndMT cells with Panc02 pancreatic cancer cells drastically enhances M2 macrophage population and tumor growth (compared with Panc02 injected alone or injected with ECs). These changes are strongly reduced with intravenously injected anti-HSP90α antibody [66]. Irradiation induces CXCR4 expression in ECs both *in vitro* and *in vivo*. This effect is abolished with p53 siRNA and in EC-p53 KO mice. The irradiation-induced CXCR4 expression induces macrophage SDF1α ^+^ recruitment and M2 polarization *in vivo* since this is inhibited with CXCR4 antagonist [67]. Consistently with these results, in human PDAC, there is a correlation between EndMT numbers and M2 macrophage infiltration. Furthermore, M2 macrophages are located close to EndMT cells [66]. All together, these data evidence that ECs undergoing osteopontin- or radiotherapy-induced EndMT induce macrophage M2 polarization in murine tumors via HSP90α secretion and CXCR4/SDF1α signaling, respectively.

#### Pericytes and perivascular mesenchymal stem cells (MSCs) induce macrophage M2 polarization

Perivascular cells regulate macrophage polarization in melanoma and pancreatic cancers. In melanoma, pericytes and MSCs influence macrophage polarization notably via MFG-E8 secretion [99]. MFG-E8, also called lactadherin, is a secreted integrin-binding protein which is overexpressed in several tumor types compared to normal tissues [100]. MFG-E8 promotes cancer progression, cancer chemoresistance, and tumor angiogenesis and is associated with poor prognosis in human melanoma. Pericytes and perivascular MSCs are the major sources of MFG-E8 secretion in melanoma tumors [85, 99]. MFG-E8 is involved in macrophage M2 reprograming since macrophage incubation with MFG-E8 induces IL-10, TGF-β, and VEGF-A secretion, and increases the proportion of CD206^+^ macrophages [101]. MFG-E8 released by apoptotic ECs or MSCs is also involved in M2 polarization [85, 101]. Indeed, *in vitro,* RAW macrophages co-cultivated with MSCs display higher M2 marker expression, which is not observed in macrophages co-cultivated with MSC MFG-E8 KO. Nonetheless, the way by which MFG-E8 induces M2 polarization still needs to be investigated. *In vivo*, MFG-E8 enhances tumor angiogenesis and tumor growth. Furthermore, higher vascularization is observed in MFG-E8 WT mice compared to MFG-E8 KO [99]. This angiogenesis enhancement is likely due to MFG-E8-induced macrophage M2 polarization. In pancreatic cancers, pericytes and CAFs are the main cells responsible for IL-33 secretion in the TME [68]. IL-33 causes M2 polarization and matrix metalloprotease-9 (MMP-9) expression in TAMs, which are mediated by the IL-33 receptor ST2 activation. MMP-9 and M2 polarization induce cancer cell intravasation and metastasis *in vivo* [68]. Furthermore, IL-33 induces TAM prostaglandin-2 secretion which enhances cancer stemness and tumor growth [84]. To conclude, perivascular cells induce TAMs M2 and pro-angiogenic phenotype via MFG-E8 and IL-33 secretion, which impacts tumor growth.

## Effects of TAMs and monocytes on tumor blood vessels

### Angiogenesis and TAMs (related to Fig. 2)

Angiogenesis refers to the formation of new blood vessels from pre-existing ones [124]. Tumor blood vessels are critical in regulating tumor growth via oxygen supply and in supporting metastasis via cancer cell dissemination. Microvessel density corresponds to the small blood vessel density in a tumor and hence is the reflection and a way to assess tumor angiogenesis [125]. It is well described that microvessel density correlates with angiogenic factors, metastasis risk, and prognosis in a huge panel of solid tumors [125, 126]. TAMs are important regulators of tumor angiogenesis [5]. Correlation between TAMs, microvessel density, and poor prognosis is observed in a lot of solid tumors. TAMs are involved in tumor blood vessel development and in the angiogenic switch [32, 127]. Indeed, in the early stage of tumor development, the vessel network development is observed several weeks earlier in CSF1-overexpressing mice than that in WT mice. TAMs promote tumor angiogenesis by pro-angiogenic factor secretion, protease secretion, and transdifferentiating themselves into vessel-like structures in a process called “vascular mimicry.”

**Fig. 2:**
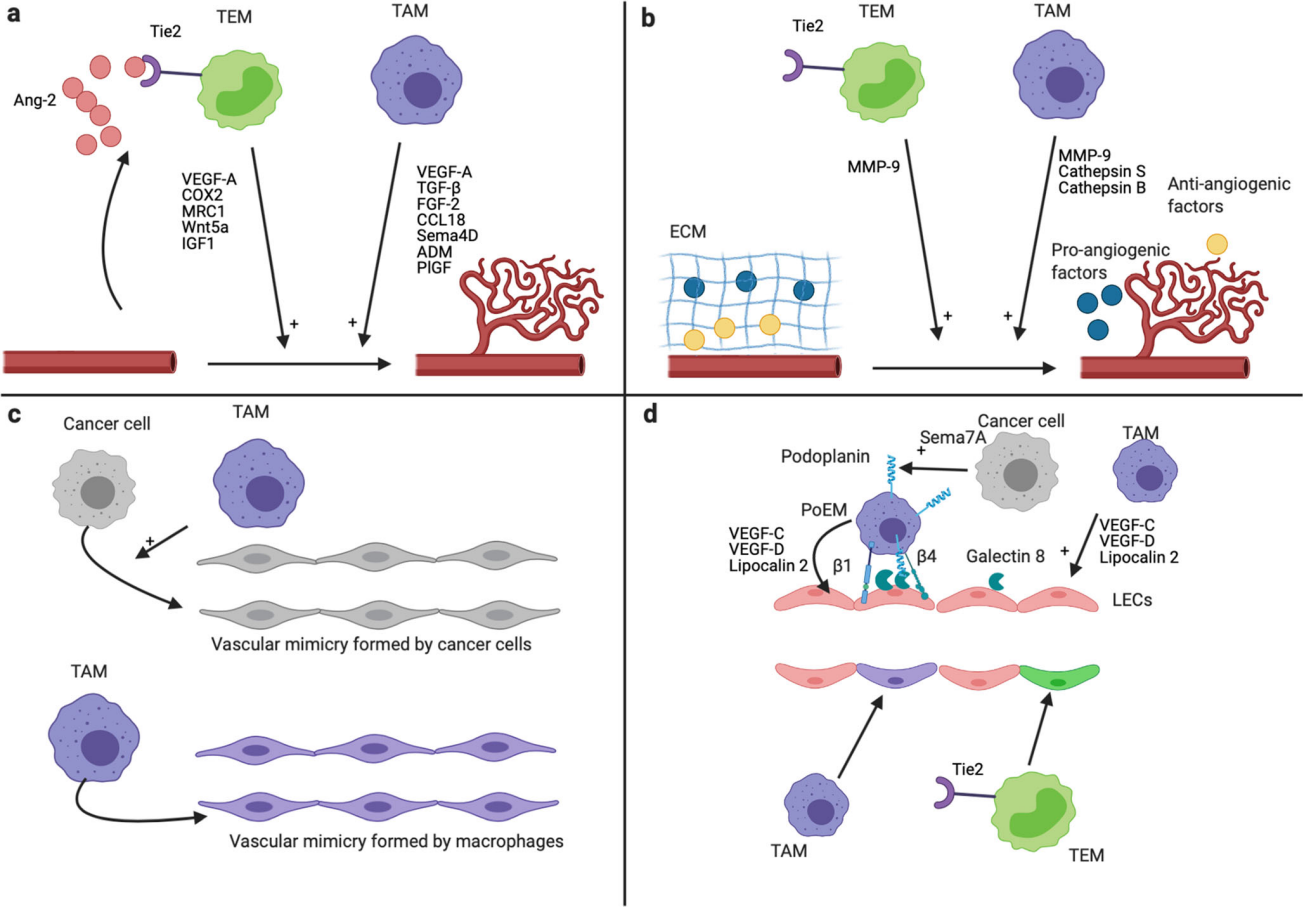
Mechanisms of tumor angiogenesis and lymphangiogenesis promotion by TAMs and TEMs. Effects of TAMs and TEMs on angiogenesis (**a**, **b**, **c**) and lymphangiogenesis (**d**). **a** TAMs and TEMs promote tumor angiogenesis via secreted factors [5]. EC-derived Ang-2 enhances the pro-angiogenic phenotype of TEMs [58, 102]. **b** TAMs and TEMs promote tumor angiogenesis via the secretion of protease. TAMs secrete MMP-9, cathepsin B, and cathepsin S, whereas TEMs secrete high level of MMP-9 [58, 103–105]. MMP-9 increases VEGF-A bioavailability via ECM degradation [106, 107]. Cathepsin S is involved in the degradation of anti-angiogenic proteins and in the formation of pro-angiogenic peptides via ECM degradation [108]. The promotion of angiogenesis by cathepsin B occurs via the induction of VEGF expression by cancer cells [109, 110]. All together, these proteases lead to an increase and a decrease of pro-angiogenic factor and anti-angiogenic factor in the TME, respectively, which promote tumor angiogenesis. **c** Upper panel: Vascular mimicry structures are perfused non-endothelial channels. They are formed by cancer cells in several cancer types, and promote tumor growth, metastasis, and angiogenesis [111, 112]. TAMs promote, at least *in vitro*, the formation of vascular mimicry channels by cancer cells [113, 114]. Lower panel: TAMs can directly form vascular mimicry structures in tumors [115]. **d** Upper panel: TAMs promote tumor lymphangiogenesis via the secretion of VEGF-C, VEGF-D, and LCN2 [116–118]. Furthermore, podoplanin-expressing macrophages (PoEMs) are able to interact with tumor LECs and are strongly involved in the promotion of tumor lymphangiogenesis [119–121]. This interaction is dependent on GAL8 (LECs), podoplanin, and β1 and β4 integrins (PoEMs) [119, 121]. The secretion of Semaphorin 7A by cancer cells promote the expression of podoplanin by TAMs [120]. Lower panel: TEMs and a subset of TAMs (called M-LECP) are able to integrate into pre-existing lymphatics, which promotes tumor lymphangiogenesis [122, 123]. This figure was created with BioRender.com

#### Pro-angiogenic factor secretion

Once in the tumor, TAMs secrete pro-angiogenic factors such as VEGF-A, TGF-β, fibroblast growth factor-2 (FGF-2), CCL18, semaphorin 4D (Sema4D), adrenomedullin (ADM), and placental growth factor (PlGF) [128–133]. Macrophage pro-angiogenic phenotype is regulated by hypoxia and lactate. Indeed, *in vitro*, conditioned media from macrophages exposed to lactate or hypoxia have higher angiogenic capacity than conditioned media from macrophages exposed to normoxia, as shown in rat corneal angiogenesis assays [134]. Hypoxia and lactate induce VEGF-A expression in macrophages via hypoxia-inducible factor-1α (HIF-1α), since this is abolished in macrophage from HIF-1α KO mice [135, 136]. It is strongly suggested in [136] that tumor-derived lactate induces TAM M2 phenotype and promotes their angiogenic phenotype. Very recently, it was shown that the expression of the lactate transporter MCT1 by macrophages is strongly involved in lactate uptake and oxidation by macrophages and in lactate-induced macrophage M2 polarization and VEGF secretion [137]. Furthermore, *in vitro*, HIF-1α and HIF-2α stability in macrophage is regulated by PI3K/Akt signaling, since HIF-1α, HIF-2α, and VEGF induction by hypoxia is strongly inhibited with PI3K inhibitors or AKT siRNA [138]. *In vivo*, TAM angiogenic phenotype and microvessel density are reduced in tumors exposed to PI3K inhibitor or in p110γ^−/−^ (a subunit of PI3K) mice. In tumors, TAMs are major VEGF producers and are located mostly in avascular and hypoxic areas [70, 136, 139]. In breast cancer, VEGF-A and TGF-β expression and secretion in TAMs are also regulated by cancer cells, notably via macrophage Fra-1 activation [132]. *In vitro*, Fra-1, VEGF-A, and TGF-β expression in macrophages from Balb/c mouse peritoneum co-cultivated with 4T1 breast cancer cells is enhanced, whereas Fra-1 siRNA diminish the enhanced VEGF-A and TGF-β expression. *In vivo*, co-injection of 4T1 and RAW macrophages subjected to Fra-1 knockdown in Balb/c mice induces tumor with less VEGF-A and TGF-β expression and with lower microvessel density than in 4T1 and RAW WT co-injected tumors [132]. FGF-2 expression and secretion in TAMs are regulated by the long non-coding RNA MALAT1. *In vitro*, MALAT1 knockdown in TAMs inhibits FGF-2 expression and secretion. MALAT1 siRNA diminishes the vascular structure formation induced by TAMs conditioned media in HUVECs and is reversed in TAMs overexpressing FGF-2 [133]. Sema4D expression and CCL18 expression in TAMs are correlated with microvascular density and these two proteins are mainly produced by TAMs [129]. *In vitro*, CCL18 induces EC tube formation via the CCL18 receptor PITPNM3 activation since this CCL18-induced tube formation is decreased in si-PITPNM3 HUVECs. Microvascular density in tumor xenografts treated with CCL18 is higher than that in the control. High angiogenesis inhibition is observed in Sema4D KO mice. The injection of WT TAMs in sema4D mice enhances angiogenesis to the same extent as that in WT mice, whereas the injection of sema4D KO TAMs does not [128]. *In vitro*, ADM secretion by macrophages is enhanced by melanoma cancer cells. TAMs promote angiogenesis via ADM secretion in ECs since these TAM conditioned media–induced angiogenesis is abolished by anti ADM. *In vivo*, colocalization between CD68^+^ RAW macrophages and ADM indicates that TAMs are a source of ADM in this melanoma murine model [131].

#### Protease secretion

TAMs also promote angiogenesis via the secretion of proteases such as cathepsins (S and B) and MMPs such as MMP-9. *In vitro*, cathepsin S and B secretion by macrophages is stimulated by the combination of M2 polarization cytokines such as IL-4, IL-10, and IL-6. This occurs in an inositol-requiring enzyme 1α (IRE1α)–dependent manner since this secretion stimulation is abolished with both IRE1α inhibitor and siRNA [140]. *In vivo*, TAMs promote angiogenesis in PDAC murine tumor model via cathepsin B and S secretion. Indeed, Rip1-Tag2 tumors inoculated with BMDMs from cathepsin B and S KO mice have a lower average vessel density than Rip1-Tag2 tumors inoculated with BMDMs from WT mice [103]. Furthermore, cathepsin S promotes angiogenesis in pancreatic Rip1-Tag2 tumors via matrix protease activity leading to an increase in pro-angiogenic factor release and in anti-angiogenic factor degradation [108]. Cathepsin B angiogenesis regulation is not fully understood but cathepsin B downregulation in multiple models leads to angiogenesis inhibition. VEGF secretion by cancer cells and in tumor is regulated by cathepsin via an unknown mechanism and could explain the positive impact of cathepsin B on tumor angiogenesis [103, 140–144]. Indeed, cathepsin B inhibition or overexpression in GBM cell lines respectively decreases or increases VEGF secretion by these cells. Furthermore, VEGF protein level is higher in breast tumor from mouse PyMT overexpressing cathepsin B than that in tumor from PyMT WT mice [109, 110, 141]. In Rip1-Tag2 pancreatic tumors, MMP-9 is involved in the angiogenic switch by the VEGF-A bioavailability enhancement [106, 107]. *In vitro*, M2 macrophages secrete high levels of MMP-9 and low levels of tissue inhibitor of metalloproteinase (TIMP)1, a MMP inhibitor, whereas M1 macrophages secrete both MMP-9 and TIMP1 [145]. Hence, cancer cells by skewing TAMs toward M2 phenotype promotes MMP-9 activity. Accordingly, M2 macrophages favor angiogenesis *in vivo* in a MMP-9-dependent manner since this ability is decreased in MMP-9 KO macrophages [145]. MMP-9 expression in macrophages is regulated by the M2 polarization marker cyclooxygenase 2 which is activated notably by MMP-1/3 and IL-6 [146, 147]. *In vivo*, tumor angiogenesis is strongly inhibited in MMP-9 KO mice or by chemical compounds inhibiting MMP-9 [104, 106]. In tumors, MMP-9 is strongly expressed in immune cells [148], mostly by neutrophils [145]. Although neutrophils constitute the major source, TAMs are also high MMP-9 producers, as shown in human colon cancer and in murine cervical cancer [104, 149]. MMP-9 expression and activity in tumors and in TAMs increase during tumor progression of the Rip1-Tag2 cancer model [104].

#### Vascular mimicry

Vascular mimicry, also called vasculogenic mimicry, refers to vascular channels formed by non-endothelial cells (mostly cancer cells). These structures were firstly described by Maniotis et al., in 1999 [150]. They showed that highly invasive melanoma cells, which notably are expressing high level of tie1, were able to form vascular perfused channels both *in vitro* and *in vivo*. Nowadays, it is known that vascular mimicry networks are also observed in numerous cancer types [111, 112, 151]. These vascular mimicry channels are perfused and connected to the general circulation. They are known to increase tumor growth and to be associated with poor prognosis and metastasis in patients [111, 112].

*In vitro*, M2 macrophages induce vascular mimicry in glioma and GBM cells [113, 114]. The macrophage-induced vascular mimicry in gliomas cells is dependent on IL-6 and COX2 induction in gliomas and GBM cells, respectively. Indeed, IL-6 expression inhibition in glioma cells and COX2 inhibition in GBM cells abolish the impact of macrophages on vascular mimicry formation by these cells. This is consistent with the fact that, in GBM patients, vascular mimicry positive areas display high TAMs infiltration. Furthermore, in glioma patients, vascular mimicry density is correlated with the quantity of M2 macrophages. In uveal melanoma, there are more macrophages in tumors having vascular mimicry than in those without vascular mimicry [152]. The correlation between macrophages and vascular mimicry appearance in tumors may be due to hypoxia since hypoxia promotes the formation of vascular mimicry as well as the infiltration of macrophages [69, 70, 153]. It would hence be interesting to investigate if TAMs induce vascular mimicry formation by cancer cells *in vivo*.

TAMs can also form vascular mimicry structures *in vitro* and *in vivo* in melanoma tumor model, in multiple myeloma, in human anaplastic thyroid carcinoma, in human meningioma, and benign melanoma tumor [115, 154, 155]. *In vitro*, exposure of multiple myeloma macrophages to VEGF and FGF-2 induces a capillary-like network associated with increased EC marker expression (factor VIII–related antigen, VE-cadherin, and VEGFR2) [154]. In the murine melanoma tumor model, these channels are functional, perfused, and connected to the vasculature since dextran is detected in these structures upon its injection in the tail vein [115]. Hypoxia is a key factor involved in vascular mimicry formation since less vascular mimicry channels are formed with HIF-1α KO macrophages [115]. Consistently with these results, in human anaplastic thyroid carcinoma, cancer cells that are closed to these macrophage channels are not necrotic or hypoxic, even at long distance from blood vessels [155]. This indicates that these channels are perfused or at least lead to tumor cell oxygenation. Additionally, vascular mimicry is observed in human malignant meningioma and benign melanoma tumors [115]. The functional significance of these TAM-derived vascular mimicry structures for tumor growth, angiogenesis, metastasis as well as for prognosis is thus worth investigating.

#### Communication with pericytes

The effects of TAMs on blood vessel angiogenesis rely on communication not only with ECs but also with pericytes. This communication between macrophages and pericytes occurs notably via Notch signaling and PDGFB-PDGFRβ-induced pericyte migration and periostin expression [156, 157]. *In vitro*, HUVEC cells co-cultivated with macrophages or pericytes enhance the formation of microvessels. Furthermore, the triple co-culture of macrophages, pericytes, and ECs is synergic in promoting angiogenesis. Notch signaling is involved in this process since Notch inhibition in each cell type inhibits angiogenesis [157]. *In vitro*, the secretion of PDGF-BB by macrophages induces pericyte PDGFRβ^+^ migration and secretion of VEGF-A and pro-angiogenic extracellular matrix component (ECM) periostin by pericytes which enhance angiogenesis [156]. The expression and secretion of PDGF-BB by macrophages are promoted by IL-4 and IL-13 but not by IL-10 [158]. The induction of PDGF-BB expression by IL-4 is mediated at least by PI3Kγ since this induction is diminished in macrophages from p110γ KO mice [159]. Accordingly, PDGF-BB expression and secretion are higher in M2 macrophages compared to M1 macrophages [158, 160]. PDGF-BB expression and secretion in macrophages are also stimulated by cancer cells. *In vitro*, macrophages exposed to U87 GBM cancer cells show higher PDGF-BB expression. This induction occurs via cat eye syndrome critical region protein 1 (CECR1) induction since it is abolished in siRNA CECR1-treated macrophages [156]. Furthermore, the stimulation of TAMs with CECR1 induces PDGF-BB expression in TAMs. Consistently with this, CECR1 expression in GBM is highly produced by TAMs and is correlated with human GBM microvascular density [156, 161]. *In vivo*, in the early steps of murine brain tumors, macrophages are involved in pericyte-endothelial interaction and thereby in tumor angiogenesis. Indeed, neural glial antigen 2 (NG2) KO specifically in macrophages strongly decreases macrophage recruitment during the beginning of murine brain tumors. Then, macrophage recruitment returns to the same level as in WT tumors in the later stages of tumor growth [162]. Interestingly, macrophage recruitment is correlated with the level of tumor blood vessel covered with pericyte and tumor angiogenesis, indicating that macrophages are most likely involved in these processes. Indeed, 10 days after the development of NG2 macrophage KO tumor, macrophage infiltration is reduced by 90% compared to WT tumors. This decrease in macrophage infiltration is associated with a lower pericyte coverage of tumor blood vessels. After 16-day tumor development, macrophage infiltration and pericyte coverage of tumor blood vessel are comparable in NG2 macrophages KO mice and in WT mice. In conclusion, TAM communication with pericytes promotes angiogenesis *in vitro*, via Notch signaling and secretion of PDGF-BB which induces pericyte recruitment. In the early steps of murine brain tumors, TAMs promote angiogenesis and the pericyte coverage of tumor blood vessels. Hence, it would then be interesting to investigate the impact of TAM-derived PDGF-BB and Notch signaling involvement in the regulation of tumor angiogenesis and pericyte recruitment in the early steps of other cancer types.

### TAMs promote tumor lymphangiogenesis (related to Fig. 2)

Tumor lymphatic vessels are formed via tumor lymphangiogenesis process and are involved in the spread of cancer cells. In tumors, VEGF-C and VEGF-D are the main factors involved in tumor lymphangiogenesis, via VEGFR3 activation in LECs. In murine tumor models, overexpression of VEGF-C/D increases tumor lymphangiogenesis. Accordingly, VEGF-C/D inhibition or VEGFR3 inhibition decreases lymph node metastasis [163]. In the TME, TAMs are major VEGF-C and VEGF-D producers [5, 116, 117]. There exists a correlation between lymphatic vessel density and VEGF-C/D production by TAMs. Furthermore, there is a correlation between TAM density, lymphatic vessel density, and lymph node metastasis in several cancers (reviewed in [39]). Recently, TAM-derived lipocalin 2 (LCN2) was observed to induce lymphangiogenesis [118, 164]. *In vitro*, TAM-derived LCN2 induces LEC proliferation, which is abolished in TAMs transfected with LCN2 siRNA. LCN2 induces lymphangiogenesis via VEGF-C expression induction in LECs, which induces VEGFR3 activation in LECs. *In vivo*, LCN2 is involved in tumor lymphangiogenesis and its associated metastases since there are less lung metastases and lower lymphatic vessel density in PyMT LCN2 KO mice than those in WT mice. Consistently with these results, LCN2 expression is correlated with lymph node metastasis in human breast and colorectal cancers [165, 166].

Other mechanisms by which TAMs promote lymphangiogenesis are by their abilities to become perilymphatic and to integrate into pre-existing lymphatics [39, 122]. These mechanisms occur in a subset of TAMs, called myeloid-lymphatic endothelial cell progenitors (M-LECP) [167]. These cells co-express macrophage markers such as CD68 (human) or CD11b (mouse) and lymphatic markers such as podoplanin and LYVE 1. These TAMs colocalize around lymphatic structures and compose macrophage-derived lymphatic structures which thereby promote lymphangiogenesis [39, 119–121]. Indeed, TAMs can transdifferentiate into LEC progenitors and acquire LEC markers such as LYVE 1 and podoplanin in murine and human tumors [39, 167, 168]. The adhesion between TAMs and LECs depends on podoplanin expression in TAMs and galectin 8 (GAL8) expression in LECs [119]. Podoplanin expression in TAMs is induced by semaphorin 7A both *in vitro* and *in vivo* during tumorigenesis and during physiological postpartum mammary gland involution [120]. Semaphorin 7A is also involved in macrophage motility, chemotaxis towards lymphatics, and TAM incorporation in lymphatics during lymphangiogenesis *in vitro* [120]. Podoplanin-expressing macrophages (PoEMs) are located near lymphatic vessels in murine breast cancer. The perilymphatic localization of PoEMs is mediated by interaction with GAL8-expressing LECs [119]. Indeed, GAL8-specific deletion in LECs or GAL8 pharmacological inhibition impairs PoEM perilymphatic localization *in vivo*. Furthermore, this interaction between PoEMs and GAL8 induces TAMs β1 integrin clustering which is needed for TAM-LEC adhesion. Another team showed that TAM location around lymphatic structures is also dependent on TAMs β4 integrin interaction with laminin 5 in murine triple-negative breast cancer [121]. Finally, PoEMs secrete high amounts of MMPs (and VEGF-C and VEGF-D) which increases VEGF-C and VEGF-D bioavailability and hence promotes lymphangiogenesis [119]. In conclusion, TAMs favor tumor lymphangiogenesis and their subsequent lymph node metastasis, either by secreted factors (VEGF-C, VEGF-D, and LCN2) or by integration of a subset of TAMs, called M-LECP, into lymphatic vessels.

### Angiogenesis and lymphangiogenesis promotion by Tie2-expressing monocytes (TEMs) (related to Fig. 2)

*In vitro*, TEMs secrete more pro-angiogenic factors such as VEGF-A, TNFα, cyclooxygenase 2, MRC1, and Wnt5a than Tie2^−^ monocytes. They are a major source of MMP-9 [58, 105]. These TEMs are recruited into tumors by EC-derived Ang-2 (see above) [56–58, 74, 77]. Furthermore, these Ang-2-activated TEMs secrete higher levels of insulin-like growth factor 1 (IGF1), cathepsin B, and thymidine phosphorylase and are more pro-angiogenic *in vitro* [58, 102]. TEMs are also pro-angiogenic *in vivo*. For example, the co-injection of glioma or ovarian cancer cells with TEMs in mice induces more vascularized tumors compared to injection of tumor cells alone or of tumor cells co-injected with CD11b^+^ myeloid cells without TEMs [102, 123, 169]. Ang-2-induced TEM IGF1 secretion induces angiogenesis and tumor growth via an IGF1 receptor–dependent activation of ECs [102]. Indeed, Ang-2-treated TEMs are more pro-angiogenic *in vitro* and *in vivo* and this increase is abolished by anti-IGF1 antibodies. Consistently with these results, the proportion of TEMs amongst total tissue TAMs is correlated with total tumor microvascular density in human ovarian, renal cell carcinoma, hepatocellular carcinoma, and non-small cell lung cancer, but not in colorectal cancer [29, 102, 170, 171]. High TEM infiltration or high number of circulating TEMs is correlated with poor prognosis in ovarian cancer and in hepatocellular carcinoma, respectively, whereas it is surprisingly correlated with good prognosis in hilar cholangiocarcinoma [102, 172–175]. These TEMs are also found in hypoxic and tumor areas enriched in small immature non-pericytic blood vessels [123, 176]. Less TAM-expressing Tie2 infiltration in murine GBM tumors is observed in HIF-1 KO mice [176]. Interestingly, in murine and human breast cancers, TEMs express lymphatic markers (e.g., LYVE 1, VEGFR-3, and podoplanin) and lymphangiogenic factors (VEGF-C and VEGF-D) and are associated with lymphatic structures [123]. These isolated breast cancer TEMs induce lymphangiogenesis both *in vitro* and *in vivo* (as shown with corneal vascularization assays) by Tie2- and VEGFR-1-dependent mechanism. Indeed, TEMs isolated from breast cancer induce lymphangiogenesis. This process is slightly inhibited by Tie2 or VEGFR inhibitors while it is abolished by the combination of both inhibitors. Interestingly, TEMs are involved in chemotherapy relapse and vessel reconstruction after chemotherapy [177]. Indeed, in murine fibrosarcoma tumors, chemotherapy (doxorubicin) firstly decreases vessel density and tumor volume which is followed by a strong increase in tumor growth and vessel density (tumor relapse). These features are correlated with TEM accumulation in the tumors. Vessel density and tumor growth promotion by doxorubicin are strongly diminished in mice with Tie2 deletion specifically in the myeloid cells. In summary, TEMs promote angiogenesis and lymphangiogenesis in several cancer types and are involved in chemotherapy relapse of murine fibrosarcoma tumors [177].

### TAMs promote metastasis (related to Fig. 3)

Metastasis process is defined as the dissemination of cancer cells from a primary tumor site into a secondary site [187]. This process is responsible for up to 90% of cancer deaths [188]. It is composed of different steps including cancer cell migration/invasion through ECM, cancer cell intravasation, cancer cell circulation and survival into the blood, cancer cell extravasation, and metastasis formation. The effects of perivascular TAMs on blood vessels are involved in cancer cell migration/invasion, intravasation, extravasation, and metastatic formation. TAM deletion in 3 different ways (CD11b^+^ TAM deletion, CSF1R mice KO, or clodronate liposomes) and monocyte recruitment inhibition into the lung by CCL2 blockade all inhibit metastatic spread from primary murine PyMT mammary tumor to lungs [16, 184].

**Fig. 3:**
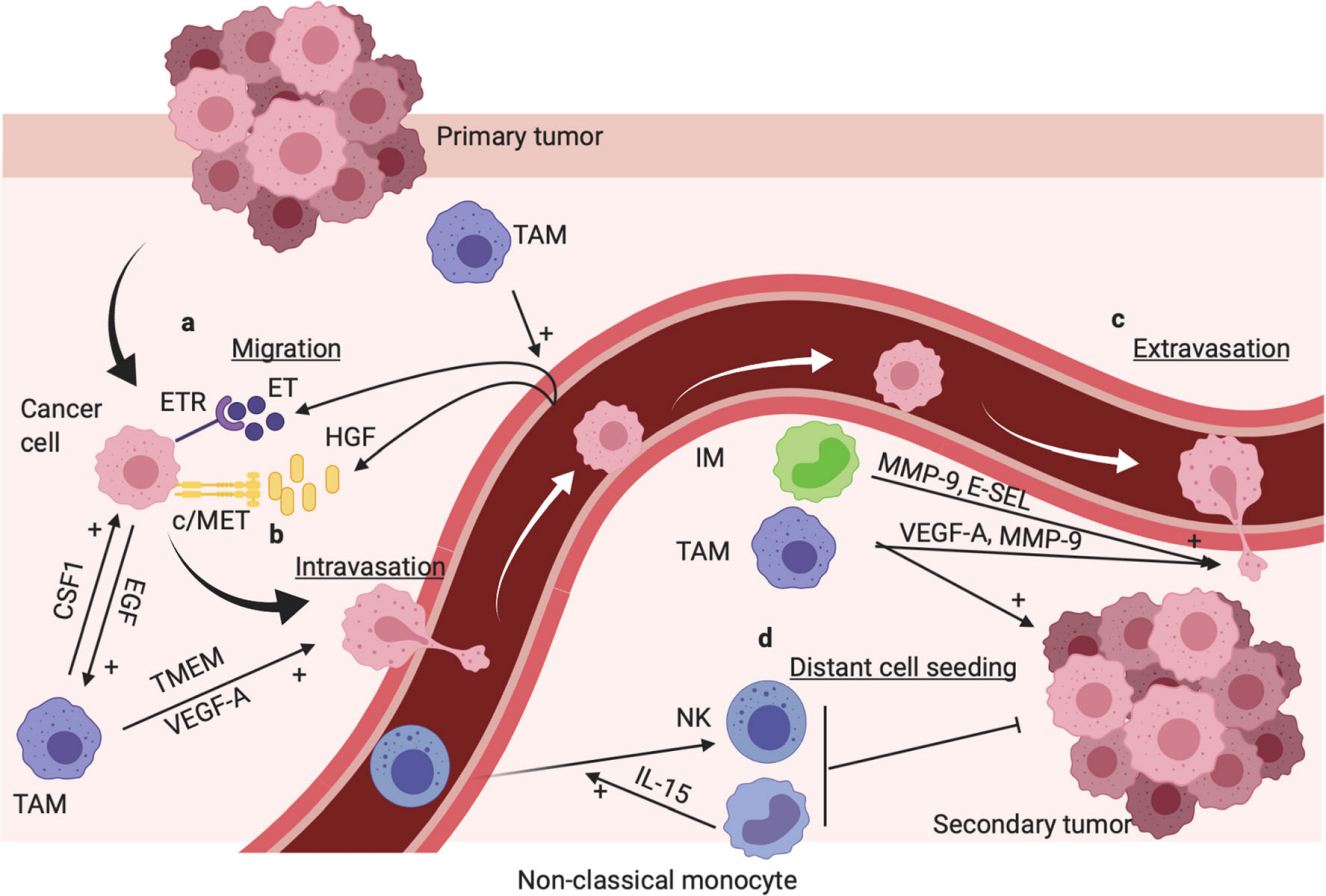
TAMs and inflammatory monocytes (IMs) promote tumor metastasis, whereas murine non-classical monocytes prevent metastasis. **a** TAMs promote cancer cell migration from primary tumor site towards blood vessel via CSF1 (TAMs) EGF (cancer cell) paracrine loop [178]. TAMs promote endothelin secretion (ET) by ECs; moreover, ET and HGF secretion by ECs induce cancer cell chemotaxis toward blood vessel via ET receptor and c-Met receptor activation, respectively [179, 180]. **b** In breast cancer, specific area called TMEM composed of a cancer cell, a TAM, and an EC promote cancer cell intravasation. Basically, TAMs induce invadopodia formation in the cancer cell which is involved in ECM breaking during EC transendothelial migration [181]. Furthermore, Tie2^high^ TAM-derived VEGF-A promote transient and local vascular leakage which favor cancer cell transendothelial migration [182]. **c** TAMs and IMs promote cancer cell extravasation notably via MMP-9 and VEGF-A-dependent vascular leakage [16, 183]. **d** TAMs and IMs promote metastasis and distant cancer cell seeding [16, 184]. Murine non-classical monocytes prevent distant cell seeding, notably via IL-15-induced NK cell recruitment [24, 185, 186]. This figure was created with BioRender.com

#### TAMs and tumor ECs promote cancer cell migration toward blood vessels

Cancer cell migration toward blood vessel is enhanced by a paracrine loop between TAMs and cancer cells, ECs and cancer cells, and the three cell types. Indeed, cancer cell migration is enhanced by perivascular TAMs involving a paracrine loop of TAMs CSF1 secretion and epidermal growth factor (EGF) secretion by cancer cells. Indeed, the inhibition of either EGF or CSF1 results in strong cancer cell migration diminution [178]. The migration of cancer cells toward blood vessels is also stimulated both *in vitro* and *in vivo* with EC-derived HGF which promotes cancer cell migration in a c-Met receptor–dependent manner [179]. Breast cancer cell motility towards HUVEC conditioned medium is impaired by cancer cell c-Met knockdown or by HUVECs HGF knockdown. Furthermore, cancer cell migration towards blood vessel is impaired by c-Met inhibition *in vivo* in breast murine cancer. A paracrine loop between TAMs, ECs, and cancer cells is involved in breast cancer cell migration/invasion toward blood vessels. Indeed, macrophage conditioned media induce ET secretion by HUVECs and ET receptor activation in cancer cells. These effects create cancer cell chemotaxis toward blood vessels which is blocked by ET-1 blocking antibody both *in vitro* and *in vivo* [180]. This paracrine loop is also responsible for tumor cell transendothelial migration and for metastasis.

#### TAMs promote cancer cell intravasation in areas called tumor microenvironment of metastasis

In breast cancer, cancer cell intravasation is enhanced in areas called tumor microenvironment of metastasis (TMEM) which is composed of one TAM, one cancer cell overexpressing the invasive isoform of “mammalian enabled” protein (Mena^INV^; an actin regulatory protein), and one EC, all three in direct contact [189]. Mechanistically, direct contact of macrophages with breast cancer cells induces Notch1-dependent Mena^INV^ expression in breast cancer cells [190]. Then, this interaction induces Rhoa GTPase–mediated invadopodia which helps cancer cells to break ECM during transendothelial migration [181]. Furthermore, VEGF-A released by Tie2^high^ TAMs enhances local and transient vascular leakiness and hence cancer cell transendothelial migration *in vitro* and *in vivo* [182]. TAM-derived TNF-α also enhances cancer cell migration toward ECs, endothelial permeability, and cancer cell intravasation in 3D fibrosarcoma and breast cancer models [191]. TAM-derived IL-1β enhances cancer cell adhesion and transendothelial migration throughout blood and lymphatic cells *in vitro* [192]. TMEM structures are observed in early tumor lesions from breast cancer of MMTV-PyMT and MMTV-HER2 mice [193]. Furthermore, in human breast cancer, TMEMs are restricted to the blood vessels (not seen in lymphatic vessels) and a high number of TMEMs are associated with increased risk of distant metastasis and correlated with breast cancer grade [194–196]. In conclusion, TAMs, as a crucial part of TMEMs, are involved in breast cancer cell intravasation and thereby involved in breast cancer metastasis. Nonetheless, since this effect of macrophages on cancer cell intravasation is restricted to breast cancer, it would be interesting to investigate if macrophages could promote cancer cell intravasation or if TMEM structures are observed in other cancer types.

#### TAMs promote cancer cell extravasation, cancer cell seeding, and distant metastasis

The extravasation step is enhanced by TAMs and monocytes, notably via blood vessel permeabilization [16, 183]. Blood vessel permeabilization is mostly promoted by TAM and monocyte VEGF-A secretion and monocyte-induced endothelial retraction in an E-selectin-dependent manner. In a 3D transmigration assay, cancer cell transmigration is diminished by 5-fold in the absence of macrophages. Interestingly, the effects of TAMs are inhibited by CCL2 blocking antibody and totally ablated in VEGF-A KO TAMs. TAM-secreted VEGF-A also enhances vascular permeability [16]. VEGF-A-induced vascular permeability is mediated by tyrosine phosphatase density-enhanced phosphatase-1 (DEP-1)–dependent Src kinase activation, which then mediates VE-cadherin uncoupling, thereby creating endothelial gaps [197, 198]. *In vivo*, VEGF-A-induced tumor vascular permeability is diminished in DEP1 KO mice or with Src inhibitor. Indeed, there is less Evans blue diffusion in healthy and tumor tissues (Miles assay) upon its injection in the tail vein of DEP-1 KO mice or in Src inhibitor–treated mice than that in WT mice or untreated mice, respectively [197, 198]. TAM-derived VEGF-A-induced vascular leakiness is a key factor involved in distant seeding of cancer cells and metastatic spread [16]. Indeed, VEGF-A deletion specifically in monocytes inhibits the efficiency of mammary cancer cell seeding in the lung, without affecting monocyte recruitment into the secondary site. Furthermore, SRC KO and DEP-1 KO mice have less metastatic spread than WT mice [198]. Vascular leakage is also enhanced by monocyte-derived MMP-9 and via monocyte-induced EC retraction in an E-selectin-dependent manner [183, 199]. In 3D *in vitro* model, monocyte MMP-9 secretion induces EC tight junction zonula occludens-1 (ZO-1) and occludin disruption, thus enhancing cancer cell extravasation [183]. Accordingly, in murine breast cancers, monocyte/macrophages are major MMP-9 producers and have a strong impact on cancer cell extravasation since MMP-9 expression and cancer extravasation are strongly reduced in tumor mice ablated of CCR2^+^ inflammatory monocytes. Moreover, in co-culture experiments, monocytes promote EC permeability and VE-cadherin dephosphorylation, which sustains cancer cell extravasation. This is dependent on monocyte interaction with EC E-selectin since this is not observed with monocytes lacking E-selectin ligands or with ECs from E-selectin KO mice. Cancer cell injection into mice induces lung vessel permeability, which depends on monocytes since this is not observed in mice depleted of monocytes. Furthermore, upon extravasation, TAMs are involved in cancer cell invasion and seeding in the ECM. Indeed, in 3D *in vitro* model, cancer cell invasion and migration are enhanced by pre-invaded macrophages [183]. Furthermore, *in vivo*, breast cancer cell pulmonary seeding is blocked by three different methods of macrophage depletion and by monocyte recruitment inhibition by CCL2 blockade [16, 184]. In conclusion, TAMs and monocytes are strong factors involved in the promotion of cancer cell extravasation, cancer cell seeding, and thereby distant metastasis.

#### Murine non-classical monocytes prevent lung metastasis

Murine non-classical monocyte (CX3CR1^high^/Ly6C^lo^) differentiation and survival depend on the orphan nuclear receptor Nr4a1, and hence, Nr4a1 KO mice have drastically less non-classical monocytes without affecting IM or macrophage population. Non-classical monocytes prevent lung metastasis formation in the PyMT breast cancer murine model or induced by LLc or B16-F10 cancer cells injected intravenously [24, 185, 186, 200]. Indeed, more lung metastases are observed in non-classical monocyte–depleted Nr4a1 KO mice upon cancer cells injected intravenously, and this is counteracted by Ly6C^lo^ monocyte injection [24]. Furthermore, PyMT mice transplanted with bone marrow from Nr4a1 KO mice show drastically more lung metastases than mice transplanted with WT bone marrow, without affecting primary tumor growth [24]. Upon cancer cell injection, non-classical monocytes interact with cancer cells in a CX3CR1-dependent manner, infiltrate the lung, engulf cancer cell material, and promote NK cell recruitment. These processes are responsible for the inhibition of lung metastases [24]. Non-classical monocyte infiltration into the lungs depends on Kindlin-3 since specific Kindlin-3 deletion in non-classical monocytes diminished their lung infiltration after cancer cell injection. This diminution is associated with an increase in lung metastases [200]. The interactions between cancer cells and non-classical monocytes and subsequent cancer cell material engulfment by non-classical monocytes depend on CX3CR1 expression in non-classical monocytes since these processes are decreased in CX3CR1 KO mice [24]. The NK cell recruitment is induced by non-classical monocytes via IL-15 secretion. Indeed, B16F-10 primary melanoma tumors induce NK cell recruitment into the lungs which is abolished with non-classical monocyte depletion or IL-15 inhibition [186]. Non-classical monocytes are high IL-15 producers, and this secretion is enhanced by primary tumors [186]. Moreover, non-classical monocytes enhance NK cell activation, notably by increasing their stimulatory receptor expression and by diminishing their inhibitory receptor expression [185]. Non-classical monocytes prevent lung metastases also via targeting exosomal content from primary tumors [201, 202]. Non-classical monocyte infiltration in lungs is enhanced by BAG6-presenting exosomes and by non-metastatic A375 melanoma cell line exosomes [201, 202].

In conclusion, several murine studies showed that murine non-classical monocytes are involved in the prevention of metastasis. Since some differences are observed between human and mouse monocytes [203, 204], it would be very interesting to confirm/correlate the results with studies performed with human monocytes.

#### TAMs promote tumor vessel abnormalization

Tumor blood vessels are abnormal, which means that they have higher permeability, less pericytes, and poor architectural network, functionality, and perfusion enhancing tumor hypoxia and acidosis. Furthermore, the decrease in blood perfusion observed in abnormal tumor vessels is responsible for the decrease in the delivery of chemotherapeutic drugs within the tumor. Tumor vessel “abnormalization” is the process by which blood vessels become abnormal. TAM promotion of vessel “abnormalization” is involved in metastasis notably by promoting cancer cell intravasation and extravasation. M2 TAMs and VEGF and PlGF secretion by TAMs are involved in the tumor blood vessel abnormalization. This abnormalization is characterized by a decrease in pericyte-covered vessels and vessel perfusion, associated with an increase in EC gaps and tumor hypoxia [130, 205]. The deletion of VEGF specifically in myeloid cells (i.e., TAMs and neutrophils) decreases tumor angiogenesis and promotes vascular normalization characterized by an increase in pericyte coverage associated with a decrease in vessel permeability. Furthermore, histidin-rich glycoprotein (HRG) drastically reduces hepatocellular carcinoma (HCC) metastasis via the inhibition of M2 TAM polarization and PlGF expression in TAMs. HRG has no effect on TAM-depleted tumors or on tumors with PlGF KO TAMs. Tumor blood vessel abnormalization and metastasis are markedly inhibited in mice with PlGF KO TAMs [130]. Interestingly, the blood vessel normalization is proposed as an emerging concept in antiangiogenic therapy since 2005 [206]. In conclusion, TAMs are strongly involved in angiogenesis induction and promotion. Blood vessels whose creation is induced by TAMs are abnormal, notably because of TAM-derived VEGF and PlGF which decrease the coverage of tumor blood vessel with pericytes, and thus promoting tumor vessel permeability and tumor metastasis.

Interestingly, the pro-metastatic activity of TAMs in hypoxia areas is regulated by metabolism. Indeed, the glycolysis and glucose uptake by TAMs are regulated by DNA damage responses 1 (REDD1). REDD1 deletion specifically in TAMs enhances the glucose uptake as well as glycolysis in TAMs. This effect induces glucose competition with ECs leading to vessel normalization and metastasis inhibition [207]. Indeed, REDD1 deletion in TAMs increases glycolytic metabolism in TAMs *in vitro*, inhibits metastasis in multiple mouse tumor models, and induces vessel normalization characterized by an increase in tumor blood vessel pericyte coverage and tumor perfusion. These effects of REDD1 deletion depend on the increase in glycolytic metabolism in TAMs since they are abolished when the increase in TAM glycolytic metabolism is abolished with the glycolytic activator PFKB3 deletion [207]. In conclusion, glucose competition between tumor ECs and TAMs regulates blood vessel features. High glucose consumption by TAMs reduces glucose availability to ECs and allows the formation of a mature and poorly metastatic vascular network. On the other hand, low glucose consumption by TAMs allows high glucose uptake by ECs which allows the formation of immature, abnormal, and pro-metastatic leaky vessels.

## Discussion

There are strong reciprocal interactions between tumor monocytes/macrophages and tumor blood/lymphatic vessels. TAMs and TEMs are involved in angiogenesis, in lymphangiogenesis, and in multiple metastasis steps, whereas blood vessels are involved in the recruitment of monocytes/macrophages/TEMs into tumors and in macrophage polarization into M2 pro-tumoral phenotype. In the last few years, many discoveries have been made about the effects of blood vessels on the polarization of macrophages, although research is still needed. IMs promote tumor growth and metastasis. Conversely, murine non-classical monocytes prevent lung metastasis, whereas human non-classical monocytes promote angiogenesis *in vitro*. Since human and murine monocytes have functional differences, it would be interesting to better understand how these monocytes prevent metastasis and to confirm that human non-classical monocytes have similar effects on tumor metastasis. Furthermore, it would be interesting to know the impact of non-classical monocytes on tumor angiogenesis *in vivo* and to better understand mechanisms regulating their infiltration into tumors. The improvement of the knowledge of the physiology of tumor blood vessels and TAMs led to the development of several therapies. Some therapy targets only TAMs with the aims to diminish TAM survival and TAM recruitment (CCL2/CCR2 or CSF1/CSF1R inhibition) or to induce a reprogramming of TAMs from M2 phenotype towards M1 phenotype [208, 209]. On the other hand, some therapies target only tumor blood vessels and aim to inhibit angiogenesis, to improve endothelial junctional integrity, to improve tumor perfusion, or to promote vascular normalization [210]. More recently, a lot of researches have been performed about the combination of anti-angiogenic drugs and immunotherapies, and some of them are currently in clinical trials (reviewed in [211, 212]). Anti-angiogenic therapies have beneficial effects on immunotherapy, and inversely. More related to this review, the combination of Ang-2 and VEGF inhibition induces the normalization of the tumor vasculature and promotes TAM reprogramming from M2 toward M1 phenotype and hence increases the M1/M2 ratio and the overall survival in sarcoma and GBM murine models [81, 213, 214]. More recently, the dual Ang-2/VEGF inhibition has been combined with CD40 or PD-1 immune therapies and showed strong synergistic effects in terms of tumor growth, overall survival, and immune cell activation in several murine tumor models [94, 215]. Interestingly, the combination of Ang-2/VEGF with PD-L1 or CD40 immunotherapies are currently in clinical trials (NCT01688206; NCT02665416). In conclusion, the improved knowledge in tumor-associated monocyte/macrophage and tumor blood vessels leads to the development of new promising and innovative therapeutic strategies which could enhance patient overall survival. Nonetheless, research on this topic is still needed in order to improve patient outcome and to diminish adverse effects of the treatments.

## Abbreviations

ADAM: a disintegrin and metalloproteinase domain–containing protein
ADM: adrenomedullin
Ang-2: angiopoietin-2
AP-1: activator protein-1
Arg-1: arginase-1
ASK1: apoptosis signal-regulating kinase 1
BMDM: bone marrow–derived macrophages
CAF: cancer-associated fibroblast
CCL: chemokine (C-C motif) ligand
CCR2: C-C chemokine receptor type 2
CD: cluster of differentiation
CSF1: colony-stimulating factor 1
CSF1R: CSF1 receptor
CTHRC1: collagen triple helix repeat containing 1
CX3CL1: chemokine (C-X3-C motif) ligand 1
CX3CR1: CX3CL1 receptor
CXCR4: C-X-C chemokine receptor type 4
EC: endothelial cell
ECM: extracellular matrix
Egfl7: EGF-like domain–containing protein 7
EMAP-II: endothelial monocyte–activating polypeptide-II
EndMT: endothelial-to-mesenchymal transition
ERK: extracellular signal–regulated kinase
ET: endothelin
FGF-2: fibroblast growth factor-2
GAL8: galectin 8
GBM: glioblastoma
HIF-1α: hypoxia-inducible factor-1α
HIF-2α: hypoxia-inducible factor-2α
HRG: histidin-rich glycoprotein
HSP90α: heat shock protein 90 α
ICAM1: intercellular adhesion molecule 1
IFIT1: interferon-induced protein with tetratricopeptide repeats 1
IGF1: insulin-like growth factor 1
IL: interleukin
IM: inflammatory monocyte
IRE1α: inositol-requiring enzyme 1α
JNK: c-Jun N-terminal kinases
KO: knockout
LCN2: lipocalin 2
LEC: lymphatic endothelial cell
LLc: Lewis lung carcinoma
LYVE 1: lymphatic vessel hyaluronic receptor 1
MCT1: monocarboxylate transporter 1
Mena^INV^: invasive isoform of mammalian enabled protein
MFG-E8: milk fat globule-epidermal growth factor 8
MHCII: major histocompatibility complex class II
M-LECP: myeloid-lymphatic endothelial cell progenitors
MMP: matrix metalloprotease
MSC: mesenchymal stem cell
mTOR: mechanistic target of rapamycin
NG2: neural glial antigen 2
NK: natural killer
p38MAPK: p38 mitogen–activated protein kinase
PDAC: pancreatic ductal adenocarcinoma
PDGF: platelet-derived growth factor
PDGFRβ: PDGF receptor β
PD-1: programmed cell death 1
PD-L1: programmed death-ligand 1
PI3K: phosphoinositide 3-kinase
PlGF: placental growth factor
PoEM: podoplanin-expressing macrophage
REDD1: regulated in development and DNA damage responses 1
SDF1α: stromal cell–derived factor 1α
Sema4D: semaphoring 4D
SOCS1: suppressor of cytokine signaling 1
TAM: tumor-associated macrophage
TCF12: transcription factor 12
TEM: Tie2-expressing monocyte
TGF-β: transforming growth factor β
TIMP: tissue inhibitor of metalloproteinase
TME: tumor microenvironment
TMEM: tumor microenvironment of metastasis
TNFα: tumor necrosis factor α
TRM: tissue-resident macrophage
VCAM1: vascular cell adhesion molecule 1
VEGF: vascular endothelial growth factor
VEGFR: VEGF receptor
WT: wild-type
ZO-1: zonula occudens-1
